# Sleep, Prospective Memory, and Immune Status among People Living with HIV

**DOI:** 10.3390/ijerph18020438

**Published:** 2021-01-08

**Authors:** Brice Faraut, Lorenzo Tonetti, Alexandre Malmartel, Sophie Grabar, Jade Ghosn, Jean-Paul Viard, Vincenzo Natale, Damien Léger

**Affiliations:** 1 VIFASOM, Vigillance, Fatigue, Sommeil et Santé publique, Equipe d’Accueil 7330, Hôtel Dieu de Paris Assistance Publique-Hôpitaux de Paris, Université de Paris, 75004 Paris, France; damien.leger@aphp.fr; 2 Centre de Référence Hypersomnies Rares, Centre du Sommeil et de la Vigilance, Assistance Publique-Hôpitaux de Paris, 75004 Paris, France; 3 Department of Psychology “Renzo Canestrari”, University of Bologna, 40127 Bologna, Italy; lorenzo.tonetti2@unibo.it (L.T.); vincenzo.natale@unibo.it (V.N.); 4 Biostatistics and Epidemiology Unit, Assistance Publique-Hôpitaux de Paris, 75004 Paris, France; malmartel.alexandre@gmail.com (A.M.); sophie.grabar@aphp.fr (S.G.); 5 Department of Family Medicine, Faculté de Médecine, Université de Paris, 75014 Paris, France; 6 Immuno-Infectiology Unit, Assistance Publique-Hôpitaux de Paris, Hôtel-Dieu, and Institut Cochin (Retroviruses, Infection and Latency), Faculté de Médecine Université de Paris, 75004 Paris, France; jade.ghosn@aphp.fr (J.G.); jean-paul.viard@aphp.fr (J.-P.V.)

**Keywords:** HIV infection, sleep, actigraphy, prospective memory

## Abstract

*Background:* Persons living with HIV (PLWH) frequently report sleep complaints, but objective measurements are still lacking regarding sleep continuity, total sleep time per 24 h, and the links with both prospective memory performance and HIV infection parameters. *Methods:* PLWH (*n* = 96) and control (*n* = 96) groups (balanced for gender and age) were monitored by 24h-actigraphy for at least seven consecutive days. The prospective memory performance was assessed through a naturalistic, activity-based task performed twice a day on the actigraph. *Results:* PLWH had greater sleep latency and worse sleep continuity (higher fragmentation index) for night-time sleep and longest daytime nap (mean duration of the longest nap). Comparable results were reported for the prospective memory task; better performance scores were associated with several sleep parameters in controls but not in PLWH. Finally, within the PLWH group, being a long sleeper per 24 h (total sleep time > 8 h including more and long daytime naps) was associated with a greater severity of the disease (lower CD4 nadir and more frequent history of AIDS-defining events). *Conclusions:* These findings indicate that PLWH have more fragmented sleep and that the severity of HIV infection is associated with increased sleep duration.

## 1. Introduction

Sleep is involved in a myriad of biological activities, all of which are critical for a balanced homeostasis of physiological functions, including immune responses [1]. Moreover, sleep deprivation triggers low-grade inflammation and immune activation [2,3,4]. The impact of sleep duration and sleep-wake rhythms on inflammation and on the immune system in human immunodeficiency viruses (HIV) infection is, however, not well established. Sleeping less than 6 hours per day has been shown to be associated with medical conditions such as diabetes and cardiovascular diseases [3,5,6]. Increased systemic inflammation has been reported in short but also in long sleepers [3,7]. In a meta-analysis, Irwin et al. found that long sleepers, i.e., people sleeping more than 8 h per day, had an increase in systemic inflammation markers which was even more pronounced than in short sleepers [7]. Although Léger et al. did not find any association between a variety of chronic diseases and long sleep, HIV infection was not specifically addressed in this study [8].

Many hypotheses exist about sleep disorders in persons living with HIV (PLWH): HIV itself could induce the production of cytokines affecting sleep when reaching glial cells [9], and such alterations of the immune system might induce circadian rhythm disruptions [10,11]. Lee et al. found an increase of fragmented sleep in PLWH with a CD4 count under 200 cells/mm^3^ [12]. Previous studies in specific populations did not find associations between insomnia and the severity of immune deficiency, but study populations were restricted to women or to veterans [13,14], and thus, may not be extrapolated to other settings. In a large HIV patient study in France, sleep disorders were associated with depression, duration of HIV infection, and combined antiretroviral treatments (cART) with efavirenz or nevirapine [15]. Overall, many uncertainties remain regarding total sleep time per 24 h and sleep continuity in HIV infection that warrant further studies with objective and validated measurements. We previously showed, in a large group of 640 PLWH, that the severity of HIV infection (lower CD4 counts) was associated with long sleep times including long naps; however, this result was based on subjective measurements [16]. Importantly, to our knowledge, only one previous investigation has assessed objective sleep parameters using actigraphy, a well establish objective measurement of sleep, in PLWH compared to a control group of healthy participants. However, a relatively small sample of 25 individuals was used, and neither total sleep time (TST) per 24 h nor the sleep fragmentation index was reported [17] (Gamaldo et al. 2013a). The data showed a longer mean sleep latency time over the 2-week monitoring period in PLWH compared to controls [17]. Here, using a relatively long period of actigraphy, we investigated the relationships between sleep duration and quality and HIV-disease characteristics in a larger sample of PLWH, compared to a control group. Hence, at least a seven-consecutive-day period of actimetry was investigated here, and the polyphasic nature of sleep (extra daytime nap episodes) was rigorously screened.

Beside sleep, we also deemed it relevant to investigate prospective memory performance through a naturalistic, activity-based task in PLWH and the control group. Prospective memory involves our ability to form and maintain an intention executed in response to a specific cue or event (here, to go to bed or to get out of bed). The PLWH group displayed a marked deficit in PM performance in the time-based task, in which a strategic process was involved, compared to an event-based task, in which spontaneous/automatic processes are involved [18]. Cognitive deficits (psychomotor performance, executive functions, memory, and attention) have been previously documented in PLWH, but few studies have examined the links with sleep [19,20]. Yet, sleep deficits have long been linked to numerous cognitive dysfunctions [21]. Previous reports have indicated associations between sleep continuity and quality with some cognitive performance tasks in PLWH [22,23]. However, these studies did not include a control group when objectively investigating sleep parameters and cognitive performance. Most of the studies used a single event-based task to evaluate prospective memory which was not always relevant to habitual tasks such as daily drug intake experienced by PLWH in their real-world environments [24].

We therefore aimed to describe the architecture and duration of the activity–rest cycle per 24 h, and also the association with prospective memory performance, assessed through a naturalistic, activity-based task in a large group of PLWH and a matched control group. Finally, we also explored the association between sleep patterns and immune parameters and disease severity in PLWH.

## 2. Methods

### 2.1. Study Design and Participants

**Figure 1:**
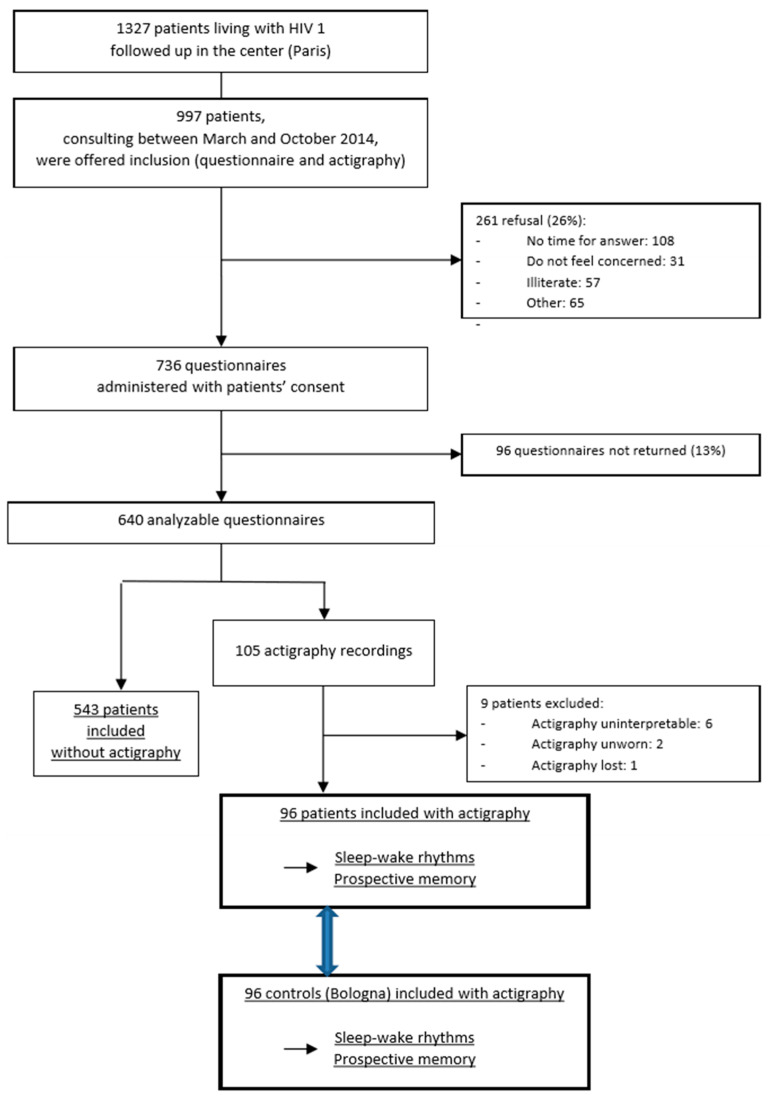
Flow chart of the study.

A ≥7-day observational study was designed in order to describe the sleep patterns of adults infected with HIV type 1. Outpatients receiving routine care at the Hotel-Dieu Hospital in Paris (France) were invited to participate in the study, and those who gave their consent were included. Patients who were actively using intravenous drugs were excluded. HIV parameters and patient characteristics, including current prescriptions (antiretrovirals and nonantiretroviral drugs), comorbidities and current conditions were evaluated with a medical questionnaire filled in by physicians between March and September 2014. Patients with acute diseases were excluded. The present actigraphic data were obtained from a subsample of a larger study [16] (Faraut et al., 2018), in which the total sleep times of patients per 24 h were objectively measured with actigraphy (see flow-chart, Figure 1). Cross-related analyses indicates that all of the sleep parameters (except sleep quality) were similar in the subsample (*n* = 96) and the larger HIV group (*n* = 640), indicating a good representativeness of the actigraphy subgroup [16]. More generally, there was good concordance for all health and socio-demographic variables between the subsample and the whole sample (see Supplementary Table S1). Sleepiness was assessed with the Epworth Sleepiness Scale (ESS) [25]. Specifically, sleep disorders were investigated using the Berlin questionnaire [26] for obstructive sleep apnea (OSA). Poor sleep quality was defined by a Pittsburgh Sleep Quality Index (PSQI) score greater than 5 using a French version of the questionnaire [27,28]. The shortened Beck Depression Inventory (BDI) score was used to assess depression [29], and both the SF-12 [30] and PROQOL-HIV [31], two validated tools, were used to assess quality of life. A total number of 96 HIV patients (26 females; 48.54 ± 11.58 years) and 96 healthy controls (HC; 26 females; 45.66 ± 14.59 year-old) were examined in this study. The HIV and control samples were balanced for gender (χ^2^ = 0; *p* = 1) and age (t_190_ = 1.52; *p* = 0.13). The healthy control group was compiled at the Laboratory of Applied Chronopsychology of the Department of Psychology “Renzo Canestrari”, University of Bologna, Bologna, Italy, using a series of previous studies [32,33,34,35] involving healthy participants. None of the participants worked flexible time schedules or night shifts, and none had complained of sleep disturbances or daytime symptoms due to unsatisfactory sleep. The exclusion criteria used in our previous studies [32,33,34,35] included sleep disorders, mental disorders, serious or acute illness, use of psychoactive medication, and disabilities interfering with or restricting mobility. For inclusion in these studies [32,33,34,35], participants had to complete the 12-item General Health Questionnaire [36], the Sleep Disorders Questionnaire [37], and the Profile of Mood States [38]; participants who did not report any sleep disorder in the Sleep Disorders Questionnaire were included if they had a General Health Questionnaire score of four or less and a Profile of Mood States score of 250 or less.

### 2.2. Actigraphy

**Figure 2:**
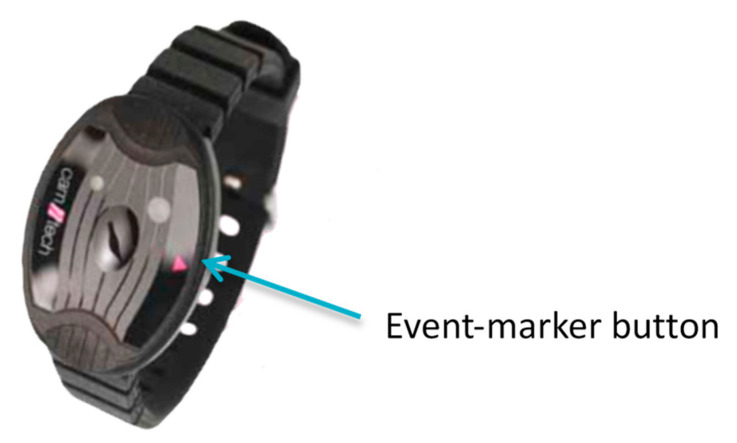
The actigraphy device.

The actigraph models Actiwatch AW64 and MotionWatch 8 (Cambridge Neurotechnology Ltd., Cambridge, UK) were used to record the sleep/wake behavior of participants (Figure 2) in 1-min epochs. Filters were set to 3 to 11 Hz. Each participant was originally requested to wear the actigraph 24-h per day around the nondominant wrist for at least six consecutive days. The mean length of actigraphy recording in PLWH was 10.99 days (SD = 1.89), while in controls, it was 8.64 days (SD = 2.89). The actigraphic records were evaluated by the same researcher (VN), setting the sensitivity to wake at a low level and using the Sleep Analysis version 5.32 software (Cambridge Neurotechnology Ltd., Cambridge, UK) [32,39].

The actigraphy device has a piezoelectric accelerometer that measures changes in acceleration and allows an indirect assessment of sleep. The activity-based, prospective memory performance test was marked as “failed” if participants failed to press the event-marker button; otherwise, it was marked as “completed” (Figure 2 shows the MotionWatch 8 Actigraph).

The following actigraphic measures of the night-time were computed:

1. Sleep start (SS): the start of sleep.
2. Sleep end (ES): the end of sleep.
3. Actual sleep time (AST): the amount of sleep.
4. Actual wake time (AWT): the amount of time spent awake.
5. Sleep latency (SL): the latency before sleep onset following bed time.
6. Sleep efficiency (SE): the percentage of time spent asleep whilst in bed.
7. Mean activity score (MAS): the average value of the activity counts per epoch over the assumed sleep period.
8. Fragmentation index (FI): the percentage of immobility phases of 1 min as a proportion of the total number of immobility phases.

Subjects reported, in a 24 h diary, every period of sleep, including the time and duration of nap episodes. Naps were actigraphically determined through the “nap analysis” function implemented within the actigraph activity and sleep analysis software, and the following actigraphic measure was computed:

- Longest nap (LN): mean duration of the longest nap.

For each participant, the mean of the actigraphic night-time and daytime measurements of the whole recording period were computed.

Patients were categorized according to their total sleep time (TST) per 24 h: below 6 h was defined as “short sleeper”, longer than 8 h was defined as “long sleeper”, and between 6 and 8 h was defined as “typical sleeper”. Objectively recorded naps were defined by a sleep episode clearly separated from the main night-time sleep episode.

### 2.3. Prospective Memory Performance

Prospective memory performance was assessed through a naturalistic, activity-based task [40]. Participants were originally requested to push the event-marker button on the top of the actigraph to signal bedtime (activity of going to bed, activity 1) and get-up time (activity of getting out of bed, activity 2). With reference to both activities, the activity-based prospective memory performance was scored as “failed” (0) if participants failed to press the event-marker button or “completed” (1) if they remembered to perform this task.

### 2.4. HIV Parameters

The main HIV parameters of interest were current and nadir CD4+ cell counts, plasma HIV RNA level, CD4+/CD8+ ratio (a surrogate marker of immune activation when <1), and a history of AIDS-defining condition (called “AIDS status”) according to the 1993 Centers for Disease Control and Prevention (CDC) clinical definition that only considers clinical events and not the CD4+ cell count. These parameters were not applicable to controls. The clinical characteristics of PLWH included in the study are reported in supplementary Table S2. The median duration of HIV infection was 16 years (interquartile range (IQR) = (6–22)), median CD4 cell count was 568 cells/mm^3^ (IQR = (454–755)) with 38% under 500 cells/mm^3^ and 37% with a CD4/CD8 ratio ≥ 1.84% had undetectable plasma HIV RNA (<20 copies/mL). AIDS status was present in 16% of patients, and 78 % had been on antiretroviral therapy in the last 10 years (median, (IQR) = (3–17)). All these clinical characteristics were similar to the larger group of PLWH from which the actigraphic subsample was extracted ([16] and see Supplementary Table S2).

### 2.5. Statistical Analysis

The day- and night-time actigraphic parameters were compared between groups through a set of independent sample t-tests, with group as the independent variable and actigraphic parameter as dependent variable. Since we performed multiple comparisons, the significance level was corrected by applying Bonferroni correction, leading us to consider *p* values less than 0.006 as significant.

For each participant, the prospective memory performance was expressed as percentage of times she/he remembered to press the event-marker button at both event 1 and event 2. The percentages were subjected to arcsin transformation in order to generate parametric statistics. We then performed a mixed ANOVA with group as the between-subjects factor (two levels: PLWH and controls) and activity-based prospective memory performance as the within-subjects factor (two levels: activity 1 at bedtime and activity 2 at get-up time).

Finally, separately for PLWH and controls, we performed a Pearson correlation analysis between the activity-based prospective memory performance at get-up time (activity 2), subject to arcsin transformation, and the actigraphic sleep parameters. Each primary criterion was analyzed with univariate logistic regressions for HIV clinical features. SAS software (version 9.4; SAS Institute, Cary, NC, USA) was used for statistical analyses (PROC MI, PROC LOGISTIC and PROC MIANALYSE).

### 2.6. Ethics

This study was approved by the Ethic Committee (Comité de Protection des Personnes Ile-de-France 1, n°2013-nov.-13404) and by the “French Commission for Data Protection and Liberties” (CNIL). Healthy controls were also requested to sign the written informed consent prior to their participation in the original studies, which were approved by the Ethics Committee in charge.

## 3. Results

### 3.1. Actigraphic Night-Time and Daytime Parameters in PLWH and Healthy Controls 

Among the eight actigraphic parameters assessed to describe sleep-wake rhythms monitoring, two displayed a highly significant differences between PLWH and healthy controls (Table 1). First, PLWH start (24:35 ± 1:23 versus 24:12 ± 1:05) and end (08:05 ± 1:25 versus 07:39 ± 1:09) their sleeping period later than controls. The time to fall asleep, 11.65 ± 8.22 min versus 7.88 ± 6.50 min, was also longer in PLWH (*p* < 0.001). Total sleep time was respectively 420 ± 61.47 min for PLWH and 416.26 ± 51.73 in the control population, while sleep efficiency was in the usual range for both groups (90.25 ± 3.60 % and 90.46 ± 3.10 %, respectively). However, the mean duration of the longest daytime nap was significantly greater in PLWH (32.49 ± 12.13 versus 22.98 ± 12.19, *p* < 0.001). More damaging, sleep continuity appeared to be affected in PLWH, with a significantly higher fragmentation index as compared to controls (*p* < 0.001).

### 3.2. Activity-Based Prospective Memory Performance in HIV Patients and Healthy Controls 

**Figure 3:**
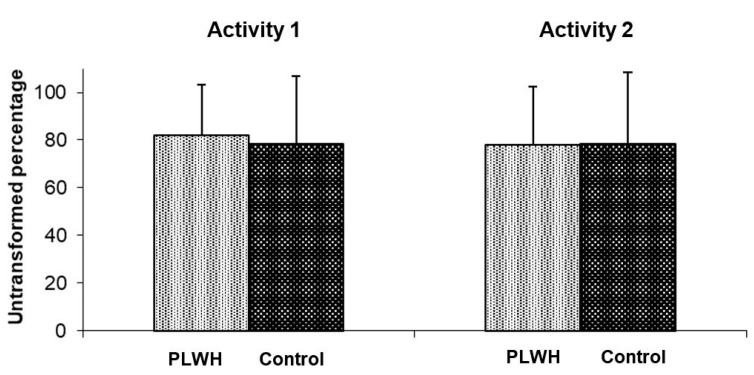
Activity-based prospective memory performance in persons living with HIV (PLWH) and controls. Interaction between within- and between-subject factors on activity-based prospective memory performance. Means and standard deviations are shown.

Prospective memory performance was assessed through the measurement of a naturalistic, activity-based task activity, i.e., remembering to push the event-marker button of the actigraph when going to or getting out of bed (activities 1 and 2, respectively). No significant difference in either activity was noticed between PLWH (79.90%; IC 95%: 75.15–84.66%) and the control group (78.41%; IC 95%: 73.65–83.16%) (F_1,190_ = 0.03; *p* = 0.86). When comparing the activities of going to bed and of getting out bed for each subject, the within-subjects factor did not reach significance (activity 1 = 80.13%, IC 95%: 76.56–83.71%; activity 2 = 78.18%, IC 95%: 74.29–82.06%; F_1,190_ = 1.50; *p* = 0.22). Finally, the interaction between the subject condition and the timing of activity was not significant (_F1,190_ = 2.29; *p* = 0.13) (Figure 3).

### 3.3. Actigraphic Night-Time Parameters and Activity-Based Prospective Memory Performance at Get-Up Time in PLWH and Controls 

As shown in Table 2, in controls, we observed significant negative correlations between activity-based prospective memory performance at get-up time (activity 2) and sleep latency, as well as mean activity score. We also observed a positive correlation between prospective memory performance and sleep efficiency. In contrast, no such significant correlations were observed among PLWH.

### 3.4. Factors Associated with Long Sleep in PLWH 

Since PLWH displayed a greater actigraphically-recorded mean duration of the longest nap than controls, we focused on the HIV group to better understand the relationship between TST per 24 h, including daytime naps and disease severity and associated immune outcomes (AIDS status, CD4 and CD8 cell counts).

We found that long sleepers practiced more daytime naps than short and usual sleepers (98 % in long sleepers versus 49 % in short and typical sleepers; OR = 20.78; (4.55–94.82)) and 74% of long sleeper practiced naps with a duration > 60 min. Long sleepers, according to actigraphy, were more likely to have a history of AIDS-defining events (OR = 4.45 (1.29–15.28); *p* < 0.01), with a lower CD4 nadir (OR = 0.66 (0.48–0.91)). No significant difference was reported in the current CD4 cell counts, but a lower CD4/CD8 ratio was associated with long sleep (*p* = 0.04; Table 3).

## 4. Discussion

PLWH frequently report sleep complaints, but objective elements are still lacking to better understand the underlying mechanisms. We report here that PLWH take longer to fall asleep, in accordance with the only previous study using a control group [17]. Our results also indicate that PLWH have a higher mean duration of the longest nap and display worse sleep continuity compared to healthy control subjects. However, other objective sleep parameters were not significantly changed, even though we observed a high rate of subjectively reported poor sleep quality in the PLWH group.

We also attempted to extend the few prior investigations on a relevant cognitive task in a larger group of PLWH. To this end, a prospective memory task was performed both frequently and routinely throughout the period of actigraphy monitoring. This task could be considered ecological plan behavior which is highly relevant to healthcare observance, such as treatment adherence. In such a habitual prospective memory task, the data showed no significant differences between PLWH and the control population. However, better scores in several sleep parameters were positively associated with performance in this prospective memory task in the control group but not in PLWH.

Finally, in the PLWH subsample monitored by actigraphy, being a long sleeper per 24 h (with a high proportion of biphasic sleepers within long sleepers) was associated with a higher severity of the disease.

### 4.1. Sleep Alterations, Immune Activation and Possible Pathophysiological Mechanisms in PLWH

The prevalence of subjectively measured sleep disturbances has already been underlined, reaching 50% of PLWH, i.e., up to five times higher than in the general population, according to recent studies [41,42]. Here, based on objective actigraphy, we report that, despite a quite similar nocturnal total sleep time, PLWH showed a greater fragmentation index during sleep periods than controls. This confirms previous polysomnography data indicating frequent nocturnal movement in PLWH [43]. These sleep alterations are still present when patients are on antiretroviral therapy [41,44].

A previous actigraphic study in a very small group of eight PLWH observed higher variability in rest–activity patterns from day to day, compared to the previously published norms in healthy volunteers [45]. Both sleep disruptions and HIV infection are possible causal factors for the genesis of an inflammatory state which is able to change sleep architecture and quality [11,46,47]. Regarding the association of naps in long sleepers with more severe immune features of the disease, we objectively confirmed with actigraphy in this subgroup of PLWH what we had previously observed in a larger group (*n* = 640) through self-reporting on sleep [16].

The mean subjective nocturnal sleep time on weekdays was 420 min (345–472) in the large HIV group (*n* = 640); this is comparable to the nocturnal sleep time reported here by actigraphy (420 ± 61.47 min in PLWH). Reports from sleep diaries in the large HIV group indicated that the proportions of short (<6 h/24 h) and long (>8 h/24 h) sleepers were 24 and 29%, respectively, including 13% of patients reporting a TST > 9 h. Interestingly, long sleepers reported more naps, and more naps longer than 1 h during the week versus short and typical sleepers (49.1% vs. 18.7% and 20.3%, and 21.1% vs. 15.5% and 4.6%, respectively; [16]. Having used an objective, reliable measure of sleep, we confirm here that these long sleepers napped twice as much as other PLWH (98% in long sleepers versus 49% in short and typical sleepers, see Table 3). We also confirm that PLWH practice longer naps than the healthy control population.

In multivariable analyses, reporting napping ≥1 h vs. non-napping was only associated with a CD4+/CD8+ ratio < 1 (OR 2.08 (1.07–4.04); *p* = 0.03) [16]. In the present study, long sleepers more frequently had a history of AIDS-defining events, a lower CD4 nadir and a CD4+/CD8+ ratio < 1 (a surrogate marker of immune activation and inflammation), markers of more severe disease. Of note, the potentially greater extent of inflammation could lead to increased sleep duration in long sleepers. This has been demonstrated in experimental animal studies, in which cerebral injection of the HIV envelope protein gp120 enhanced the expression of pro-inflammatory cytokine, e.g., IL-1, and lengthened nonrapid eye movement sleep [48]. In humans, the association of inflammatory status (assessed by C-reactive protein (CRP)) with napping frequency in relation to night-time sleep was investigated in 2147 young adults; it was found that one subject who napped every day within the long sleep (>9 h of night-time sleep) group had elevated CRP levels [49].

Hence, long sleepers (a population with a higher rate of long naps) and PLWH napping ≥ 1 h are associated with CD4+/CD8+ ratio < 1, meaning that these patients are at higher risk of immuno-inflammation. This suggests potential similar mechanism(s) as reported in experimental studies, i.e., that this higher inflammation status could lead to enhanced total sleep time in the PLWH identified as long sleepers. Furthermore, the higher frequency of long naps could be seen as a marker of general health degradation, as suggested by a higher history of AIDS-defining events (a higher vulnerability to opportunistic infections). As such, these long naps could be understood as passive sleep episodes (and not as scheduled countermeasures to compensate a sleep debt) due to extreme fatigue, potentially alerting a deterioration in the health of PLWH. Poor sleep quality is highly prevalent in this population (see supplementary Table S1), and may be the first stage toward longer total sleep time and long daytime naps, considering that increased time in bed does not improve sleep, but rather, promotes sleep fragmentation.

In addition, anxio-depressive disorders could partly explain the greater sleep fragmentation measured in PLWH. Indeed, in the present actigraphy study, 39% of PLWH (similar to the proportion in the original larger group; [16]) were affected by minimal to severe self-reported depressive symptoms (see Supplementary Table S1). Moderate or severe depression has previously been associated with poor sleep quality (PSQI > 5) in multivariable analyses in our large cohort [16]. Previous studies have indicated similar prevalences: 40% displayed depressive symptoms in a Chinese study, and 17 to 47% described depression and 22–49 % anxiety in a large meta-analysis in the UK population [42,50].

### 4.2. Cognitive Performance, Prospective Memory, and Sleep in PLWH

Damage to the neural system caused by, e.g., inflammatory responses to HIV infection, could affect executive functions. Indeed, uncontrolled HIV infection damages the immune system. However, antiretroviral treatments (cART) improve life expectancy in HIV patients, who now have similar mortality rates to the general population provided that the CD4 cell count remains over 500/mm^3^ with a sustained suppression of plasma HIV RNA [51]. The side effects of antiretroviral combinations or individual drugs such as zidovudine and efavirenz, including psychiatric diseases [52], disturbed sleep and unusual dreams [53], or obstructive sleep apnea (OSA) [54] caused by weight gain [55,56], have been reported and could favor both sleep disruptions and cognitive deficits. These treatments have been reported to change sleep architecture and spectral profile with reduced time to sleep onset and lower performance in attention tasks [57,58].

Prospective memory could be seen as our “remember to remember” skill, which is highly significant when it means remembering to take medication. A prospective memory task has been described as follows: (1) formation of the intention to defer an action; (2) retention of the deferred action over a delay interval; (3) initiation of the deferred action upon detecting the appropriate cue (in the present study to push the event-marker button on the top of the actigraph aiming to signal bedtime and get-up time); and (4) execution of the deferred action [59]. Our prospective memory paradigm can be considered as a routine everyday task which may be more relevant to clinical populations for which healthcare actions needs to be made regularly, e.g., in patients on cART, in whom deficits in neurocognitive functions are frequently reported [60]. Sleeping after encoding appears to influence and strengthen the memory to perform an intended action at a planned time, suggesting a beneficial effect of sleep on prospective memory [61,62], although this point is still being debated [40,63]. Reduced sleep quality and quantity are connected to altered and diminished encoding and memory consolidation abilities over time [23,64].

Better self-reported—as opposed to objectively measured—sleep quality (assessment of the quality of five dimensions of sleep within the last 30 days, i.e., satisfaction with sleep; alertness during waking hours; timing of sleep; sleep efficiency; and sleep duration), has been correlated with better scores regarding distinct cognitive tasks (verbal learning and visual memory; [65]). We should mention that our subsample monitored by actigraphy had worse subjective sleep quality variables according to PSQI (see Supplementary Table S1), which could evoke a high level of anxiety and could also partly explain the absence of expected correlations found here between the sleep variables and the prospective memory performance in PLWH, in contrast to what was observed in controls.

However, the fact that better scores for several sleep parameters (sleep efficiency and latency) were positively associated with this prospective memory task in healthy controls but not in PLWH suggests that sleep quality is not the main factor of memory performance [40,63]. Indeed, the deteriorated fragmentation index in PLWH was not correlated with any performance index of the task. The quantity of sleep, which was comparable in PLWH and controls, could be more critical in the performance of this performance task. Alternatively, additional nonsleep-dependent mechanisms could be correlated to the performance level.

Indeed, as previously mentioned, it has been observed that PLWH present a more marked deficit in prospective memory performance in time-based tasks (i.e., involving strategic processes) compared to event-based tasks (i.e., involving spontaneous/automatic processes) [18]. A study confronting objective sleep assessments and cognitive performance was performed in a group of PLWH, virologically controlled on cART, who were recorded for a single night using polysomnography, and then monitored for two weeks with actigraphy. Better results at some of the tasks of the cognitive performance battery (attention and psychomotor speed) were associated with several polysomnographic parameters such as higher total sleep time, sleep efficiency, and reduced waking after sleep-onset (WASO) [22]. However, these investigations were conducted in a small group of PLWH (*n* = 36), were based on only one night of polysomnography, did not include prospective memory performance assessments, and there was no control group. Furthermore, the associations no longer held true when the same sleep parameters were assessed with the data of the two-week actigraphy [22]. Here, based on actigraphy recordings, we found that better prospective memory performance was associated with greater sleep efficiency in the control group but not in PLWH, in accordance with the study of Gamaldo et al. [22].

### 4.3. Limitations

The strengths of this study lie in the relatively large size of our well-characterized group of PLWH, the comparison to a control group, and the assessments of a real-world functioning prospective memory task and objective sleep monitoring. Still, the current investigation also had several limitations that warrant consideration. In the present study, when compared to a control population, prospective memory performance was not significantly different in the two groups. It is possible that a longer period of investigation or performing a more complex task could have revealed differences.

Additionally, the HIV-positive and -negative groups were well matched with regards to sex and age, but were recruited from two distinct European sleep centers (Paris Hôtel-Dieu and Bologna University) with a potential bias in the understanding of the instructions given by different investigators for the naturalistic, activity-based task. This may have influenced the level of adherence to the prospective memory performance task.

Also, in HIV^+^ patients, the absence of a predictable association between better activity-based, prospective memory and better sleep parameters (higher sleep efficiency, shorter sleep latency) measured in healthy participants suggests additional non sleep- dependent mechanisms involved in the performance of HIV patients which are not discussed here. Possibly, among the HIV^+^ group, a greater heterogeneity in cognitive abilities than in the control group could partially explain this result. Alternatively, as mentioned earlier, the critical sleep factor for the level of prospective memory performance could be total sleep time, which was similar in both groups. Finally, polysomnographic recordings would have allowed us to explore the potential role of sleep architecture in prospective memory performance. Indeed, recent studies have highlighted the potential role of slow wave and REM sleep in prospective memory performance [66,67] (Leong et al., 2019; Scullin et al., 2019).

We also must acknowledge that a potential bias could explain why long sleepers had worse immune statuses compared to short and typical sleepers (Table 3), i.e., patients presenting a worse immune status may be less active; therefore, there was a higher likelihood that their sedentary activity was incorrectly scored by actigraphy, due to its low specificity, as sleep.

## 5. Conclusions

Overall, these findings indicate that PLWH have more fragmented sleep, and that the severity of HIV infection is associated with the lengthening of sleep duration. Actigraphy may be a valuable tool for the identification of these kinds of sleep troubles in clinical settings. The gold standard, i.e., polysomnography monitoring, although experimentally difficult to achieve over a large number of recording days and with many patients, is required to confirm the original and new associations described here between long sleepers and nappers and unfavorable immune and health conditions in HIV patients. Finally, longitudinal studies collectively assessing cognitive responses, objective sleep, and immune status are required to better define the long-lasting effects of sleep quality, architecture, and amount on neurocognitive condition during the progression of chronic illnesses such as AIDS.

## Figures and Tables

**Table 1:** Actigraphic night-time and daytime parameters in persons living with HIV (PLWH) and controls.

	PLWH	Controls	Statistics	
	Mean ± SD (IC 95%)	Mean ± SD (IC 95%)	t_(190)_	*p* ^a^
**Night-time parameters**				
SS	24:35 ± 1:23 (24:18–24:52)	24:12 ± 1:05 (23:59–24:25)	2.15	0.03
ES	08:05 ± 1:25 (07:47–08:22)	07:39 ± 1:09 (07:25–07:53)	2.31	0.02
AST	420 ± 61.47 (407.55–432.45)	416.26 ± 51.73 (405.77–426.74)	0.46	0.65
AWT	29.88 ± 13.45 (27.16–32.61)	31.19 ± 11.74 (28.81–33.57)	−0.72	0.47
SL	11.65 ± 8.22 (9.98–13.32)	7.88 ± 6.50 (6.57–9.20)	3.52	**<** **0.001**
SE	90.25 ± 3.60 (89.52–90.98)	90.46 ± 3.10 (89.83–91.09)	−0.42	0.67
MAS	13.40 ± 5.76 (12.23–14.57)	13.79 ± 5.29 (12.72–14.87)	−0.49	0.62
FI	32.83 ± 8.58 (31.09–34.57)	27.84 ± 7.40 (26.34–29.34)	4.32	**<** **0.001**
**Daytime parameter**				
LN	32.49 ± 12.13 (30.03–34.95)	22.98 ± 12.19 (20.51–25.45)	5.42	**<0.001**

Notes: SS = sleep start (h:min); ES = sleep end (h:min); AST = actual sleep time (min.); AWT = actual wake time (min.); SL = sleep latency (min.); SE = sleep efficiency (%); MAS = mean activity score (activity counts); FI = fragmentation index (sum of percentages); LN = mean duration of the longest nap (min.). ^a^ Bonferroni corrected *p*-values. Means and standard deviations (SD) of actigraphic night-time and daytime parameters of PLWH and controls are reported together with statistics. Significant effects are in bold type.

**Table 2:** Actigraphic night-time parameters and activity-based prospective memory performance at get-up time in persons living with HIV (PLWH) and controls.

	PLWH	Controls
	PM Performance	PM Performance
**Night-time parameters**		
SS	−0.13	0.08
ES	−0.15	−0.11
AST	−0.02	−0.17
AWT	−0.09	−0.20
SL	−0.06	−0.28 **
SE	0.10	0.26 *
MAS	−0.14	−0.21 *
FI	−0.01	−0.13

Notes: SS = sleep start; ES = sleep end; AST = actual sleep time; AWT = actual wake time; SL = sleep latency; SE = sleep efficiency; MAS = mean activity score; FI = fragmentation index; HIV = human immunodeficiency virus; HC = healthy controls; PM = prospective memory. * *p* < 0.05, ** *p* < 0.01. Pearson’s correlations between actigraphic night-time parameters and activity-based prospective memory (PM) performance at get-up time in PLWH and controls. Significant correlations are in bold type.

**Table 3:** Factors associated with long sleepers in persons living with HIV.

	Long Sleepers *n* (%) or Mean (sd) *n* = 41	Short and Typical Sleepers *n* (%) or Mean (sd) *n* = 55	OR (IC 95%) (*n* = 96)	*p*
**AIDS status**	11 (27)	5 (9)	4.45 (1.29–15.28)	**<0.01**
**Log_2_(Nadir CD4)**	7.75 (7.22)	8.26 (7.23)	0.66 (0.48–0.91)	**0.01**
**CD4 cell count***n* (%)				
≥500 cells/mm^3^	27 (64)	36 (65)	1	
<499 cells/mm^3^	15 (36)	19 (35)	1.08 (0.46–2.53)	0.86
**CD4/CD8 ratio < 1**	30 (71)	29 (54)	2.37 (1.00–5.62)	**0.04**
**Daytime napping**	41 (98)	27 (49)	20.78 (4.55–94.82)	**<0.01**

Each primary criterion was analysed with univariate logistic regressions.

## Data Availability

All datasets generated for this study are included in the article.

## Supplementary Materials

The following are available online at https://www.mdpi.com/1660-4601/18/2/438/s1, Table S1: Cross-reference table between the actigraphy subgroup and the whole PLWH cohort explored with self-reported questionnaires; Table S2: Clinical characteristics of persons living with HIV (PLWH) included in the actigraphy study.
