# Supplementary material for: Sleep, Prospective Memory, and Immune Status among People Living with HIV
Source: Int J Environ Res Public Health. 2021 Jan 8;18(2):438. doi: 10.3390/ijerph18020438 (PMC7826879; doi:10.3390/ijerph18020438)
Supplement: Supplementary file 1 [file ijerph-18-00438-s001.pdf]

Supplementary Material:

Table S1. Cross-reference table between the actigraphy subgroup and the whole PLWH cohort explored with self-reported questionnaires. Significant differences are in bold type.

	Missing values	Total (n=640)	Actigraphy (n=96)	No actigraphy (n=543)	p
**Age** median (IQR)	0	48 (40-55)	48 (40-55)	47 (39-55)	0.67
**Men** n (%)	1	463 (72)	70 (73)	393 (72)	0.91
**Body Mass Index** n (%)	0				**0.049**
Underweight (< 18)		67 (11)	8 (8)	59 (11)	
Normal (18–25)		354 (55)	45 (46)	309 (57)	
Overweight (25–30)		165 (26)	36 (37)	129 (24)	
Obese (> 30)		54 (8)	8 (8)	46 (8)	
**Professional activity** n(%)	12	428 (68)	61 (64)	367 (69)	0.29
**Years since HIV diagnosis** median (IQR)	1	15 (7-22)	16	14 (8-22)	0.75
**History of AIDS-defining condition** n (%)	8	114 (18)	16 (17)	98 (18)	0.67
**Current treatment** n (%)	0				0.20
2 NRT1+1 NNRTI		241 (38)	31 (32)	210 (39)	
2 NRT1+1 protease inhibitor		152 (24)	30 (31)	123 (23)	
2 NRT1+1 integrase inhibitor		63 (10)	12 (12)	51 (9)	
Other		183 (29)	24 (25)	159 (29)	
**Viral load <20 copies/ml** n (%)	8	500 (79)	81 (84)	419 (78)	0.17
**CD4 cell count nadir (cells/mm3)** median (IQR)	17	252 (137-360)	253 (146-383)	252 (137-356)	0.83
**CD4 cell count (cells/mm3)** median (IQR)	1	596 (450-805)	568 (454-755)	696.5 (450-814)	0.46
≥ 750 n (%)		193 (30)	25 (26)	168 (31)	
500-749 n (%)		232 (36)	38 (39)	194 (36)	
350- 499 n (%)		138 (22)	25 (26)	113 (21)	
< 349 n (%)		76 (12)	9 (9)	67 (12)	
CD4 < 200 n (%)		15 (2)	3 (3)	12 (2)	
**CD4/CD8 ratio ≥ 1** n (%)	8	262 (41)	37 (39)	225 (42)	0.53
**PROQOL-HIV global (Life quality)** median (IQR)	181	77 (62-85)	78 (62-86)	77 (62-85)	0.80
**SF-12** median (IQR)	76				
SF-12 physical component scale		53 (46-57)	52 (57-46)	54 (46-57)	0.53
SF-12 mental component scale		45 (35-53)	44 (33-51)	45.5 (35-53)	0.30
**Beck Depression inventory** n (%)	158				0.90
No depression		302 (63)	48 (62)	254 (63)	
Minimal depression		102 (21)	16 (21)	86 (21)	
Moderate depression		68 (14)	13 (17)	55 (14)	
Severe depression		10 (2)	1 (1)	9 (2)	
**PSQI >5 (poor sleep quality)** n (%)	219	288 (68)	59 (87)	229 (65)	**<0.001**
**High risk of obstructive sleep apnoea (Berlin score ≥ 2)** n (%)	207	91 (21)	18 (25)	73 (20)	0.36
**Epworth sleepiness scale** n (%)	71				0.25
No sleepiness (0-10)		339 (60)	51 (58)	288 (60)	
Sleepiness (11–15)		189 (33)	27 (31)	162 (34)	
Severe sleepiness (> 16)		41 (7)	10 (11)	31 (6)	
**Chronotype** n (%)	1				0.87
Morning		183 (29)	26 (27)	157 (29)	
Evening		206 (32)	31 (32)	175 (32)	
Intermediate		250 (39)	40 (41)	210 (39)	
**Short Sleeper (<6h)** n (%)	31	149 (24)	26 (29)	123 (24)	0.45

**Typical Sleeper (6-8h)** n (%)		285 (47)	37 (42)	248 (48)	
**Long sleeper (>8h)** n (%)		175 (29)	26 (29)	149 (29)	
**Total sleep time /night (week)** median median (IQR)	31	7:00 (5:45-7:52)	7 :15 (5:35-7:55)	7:00 (5:50-7:51)	0.82
**Total sleep time / 24h (week)** median (IQR)	31	7 :15 (6:00-8:09)	7 :20 (5:40-8:04)	7 :15 (6:00-8:09)	0.48
**Total sleep time /night (week-end)** median (IQR)	86	7:55 (6:30-8:50)	7 :54 (6:15-8:44)	7 :55 (6:30-8:50)	0.48
**Total sleep time / 24h (week-end)** median (IQR)	86	7:55 (6:30-8:50)	7:54 (6:15-8:45)	7:55 (6:30-8:50)	0.46
**Nap/ week (n)** median (IQR)	1	0 (0-1) /1	0(0-1)	0 (0-2)	0.11

Table S2. Clinical characteristics of persons living with HIV (PLWH) included in the actigraphy study.

	**PLWH (n=96)**
**Years since HIV diagnosis** median (IQR)	16 (6-22)
**History of AIDS-defining condition** n (%)	16 (17)
**Anti-retroviral therapy** n (%)	76 (78)
**Anti-retroviral therapy duration** Year (median (IQR))	10 (3-17)
**Current treatment** n (%)	
2 NRT1+1 NNRTI	31 (32)
2 NRT1+1 protease inhibitor	30 (31)
2 NRT1+ 1 integrase inhibitor	12 (12)
Other	24 (25)
**Viral load <20 copies/ml)** n (%)	81 (84)
**CD4 cell count nadir (cells/mm3)** Median (IQR)	253 (146-383)
**CD4 cell count (cells/mm3)** median (IQR)	568 (454-755)
≥ 750 n (%)	25 (26)
500-749 n (%)	38 (39)
350- 499 n (%)	25 (26)
< 349 n (%)	9 (9)
< 200 n (%)	3 (3)
**CD4/CD8 ratio ≥ 1** n (%)	37 (39)

NRTI nucleoside reverse transcriptase inhibitor, *NNRTI* non-nucleoside reverse transcriptase inhibitor.
